# Social Vulnerability and Risk of Suicide in US Adults, 2016-2020

**DOI:** 10.1001/jamanetworkopen.2023.9995

**Published:** 2023-04-26

**Authors:** Shuhan Liu, Samuel B. Morin, Natalie M. Bourand, Isabella L. DeClue, Gustavo E. Delgado, Jiahe Fan, Sabrina K. Foster, Maaz S. Imam, Coulter B. Johnston, Franklin B. Joseph, Yihao Lu, Ujjwal Sehrawat, Li Chun Su, Ketaki Tavan, Kelly L. Zhang, Xingruo Zhang, Loren Saulsberry, Robert D. Gibbons

**Affiliations:** 1 The University of Chicago, Chicago, Illinois; 2 Institute for Population and Precision Health, The University of Chicago, Chicago, Illinois; 3 Harris School of Public Policy, The University of Chicago, Chicago, Illinois; 4 Department of Public Health Sciences, The University of Chicago, Chicago, Illinois; 5 Department of Medicine, The University of Chicago, Chicago, Illinois

## Abstract

**Importance:**

There were over 45 000 suicides in the US in 2020, making suicide the 12th leading cause of death. If social vulnerability is associated with suicide rates, targeted interventions for at-risk segments of the population may reduce US suicide rates.

**Objective:**

To determine the association between social vulnerability and suicide in adults.

**Design, Setting, and Participants:**

This cohort study analyzed 2 county-level social vulnerability measures (the Social Vulnerability Index [SVI] and the Social Vulnerability Metric [SVM]) and US Centers for Disease Control and Prevention–reported county-level suicides from 2016 to 2020. Data were analyzed November and December 2022.

**Exposures:**

County-level variability in social vulnerability.

**Main Outcomes and Measures:**

The primary outcome measure was number of county-level adult suicides from 2016 to 2020, offset by county adult population during those years. The association between social vulnerability (measured using the SVI and the newly created SVM for 2018) and suicide was modeled using a bayesian-censored Poisson regression model to account for the CDC’s suppression of county-level suicide counts of less than 10, adjusted for age, racial and ethnic minority, and urban-rural county characteristics.

**Results:**

From 2016 to 2020, there were a total of 222 018 suicides in 3141 counties. Comparing the least socially vulnerable (0% to 10%) to the most socially vulnerable (90% to 100%) counties, there was a 56% increase in suicide rate (17.3 per 100 000 persons to 27.0 per 100 000 persons) as measured by the SVI (incidence rate ratio, 1.56; 95% credible interval, 1.51-1.60) and an 82% increase in suicide rate (13.8 per 100 000 persons to 25.1 per 100 000 persons) as measured by the SVM (incidence rate ratio, 1.82; 95% credible interval, 1.72-1.92).

**Conclusions and Relevance:**

This cohort study found that social vulnerability had a direct association with risk for adult suicide. Reducing social vulnerability may lead to life-saving reduction in the rate of suicide.

## Key Points

**Question:**

Is social vulnerability associated with suicide rates for US adults?

**Findings:**

In this cohort study including 222 018 suicides in 3141 US counties, high scores on both the US Centers for Disease Control and Prevention’s Social Vulnerability Index and the new Social Vulnerability Metric were associated with higher adult suicide rates at the county level. Comparing the lowest 10% and highest 10% in the indices showed a 56% higher suicide rate for the Social Vulnerability Index and 82% higher for the Social Vulnerability Metric.

**Meaning:**

These results suggest a possible quantitative approach to reduce suicide rates through targeted interventions of social vulnerability.

## Teaser

This cohort study using county-level US Census data examines the association of social vulnerability with suicide rates using 2 indices from the US Centers for Disease Control and Prevention.

## Introduction

There were over 45 000 suicides in the US in 2020, making suicide the 12th leading cause of death.^1^ Suicide rates were 30% higher in 2020 than they were in 2000, highlighting the importance of a targeted suicide risk prediction model.^1^ Studies have shown that social ecological factors are associated with suicide rates. These include environmental factors such as air pollution^2^ and community factors such as youth exposure to violence and crime,^3^ access to quality health and mental health care,^4,5,6^ food insecurity,^7^ and employment status.^8^ These ecological factors are indicators of social determinants of health (SDOH), which are an array of nonmedical influences on health outcomes.^9^ More specifically, there is increasing awareness that challenging issues within a person’s community contribute to suicide risk.^10^ These factors include lack of access to health care, stress of acculturation, exposure to community violence, historical trauma, and discrimination. Broader societal risk factors include stigma associated with help-seeking and mental illness, easy access to lethal means of suicide among people at risk, and unsafe media portrayals of suicide.^10^ The impact of social vulnerability on suicide has been shown to be pervasive across the world. In Australia from 2010 to 2021, age-standardized suicide rates were highest in areas with the lowest socioeconomic status,^11^ and suicide rates increased significantly for those living in the most socioeconomically disadvantaged areas (2009 to 2016), with little changes in less socioeconomically disadvantaged areas.^12^ In Germany,^13^ suicide rates were inversely associated with income, educational attainment, and positively associated with unemployment rates and social isolation (ie, living alone). Social isolation moderated the decreases in suicide rates associated with higher income. In South Korea,^14^ suicide rates were positively associated with individuals aged over 65 years, vacant homes in the area, divorce rates, single elderly households, detached houses, smoking, and obesity. The effect sizes were larger among males and in urbanized areas. In the US,^15^ suicide rates were highest in rural areas and increased from 1999 to 2016 more rapidly relative to large metropolitan areas. Access to guns (ie, gun shops) was associated with increased suicide risk at the county level. High social fragmentation (eg, single-person households, percentage of unmarried residents), low social capital (eg, number of charities, arts, and nature facilities, religious organizations), high area deprivation (eg, education, occupation, employment, income), and lack of medical insurance were all associated with increased suicide risk at the county level.

This study addresses several gaps in the research on suicide. First, our focus is on measures of social vulnerability, the CDC Social Vulnerability Index (SVI)^16^ and the Social Vulnerability Metric (SVM),^17^ which integrate across several individual, yet intercorrelated, social domains. The SVI is a traditional measure consisting of a sum of items measuring sociodemographic factors and which provides a national percentile for each community (at the county level). By comparison, the SVM is a model-based measurement device based on multidimensional item response theory that differentially weights the items in terms of their ability to discriminate high and low levels of social vulnerability and the difficulty in achieving the item, effectively constructing an overall Bayes estimate of social vulnerability for each community (zip code or county). Second, in our statistical analysis of the data we have provided a rigorous approach to dealing with the CDC’s censoring of county-level suicide rates (any county with fewer than 10 suicides is censored), which they use for privacy protection. Third, we hypothesize that increased social vulnerability will be associated with higher suicide risk in US adults and that the inclusion of race in the SVI measure might produce biased estimates of the association between social vulnerability and suicide. As we demonstrate, the SVM in particular is an important new tool for incorporating social vulnerability into the development of suicide risk prediction systems and targeting interventions to reduce suicide risk.

## Methods

This study was reviewed by the University of Chicago institutional review board and was determined to be exempt from informed consent requirements as it relied on publicly available data. This report follows the Strengthening the Reporting of Observational Studies in Epidemiology (STROBE) reporting guidelines for observational studies.

### Measures

We used 2 measures of social vulnerability, the SVI^16^ and the SVM.^17^ The SVI ranks US counties in terms of their social vulnerability on 17 SDOH indicators drawn from the domains of education access and quality, health care and quality, neighborhood and built environment, social and community context, and economic stability.^16^ The SVI was designed as a relative measure of the degree to which a community is at risk during a public health emergency, highlighting the needs of socially vulnerable populations in emergency response and recovery efforts. The SVM was developed to provide a zip code–level or county-level social vulnerability measure based on multidimensional item response theory applied to the domains of demography, education, economic context (eg, unemployment rate), physical infrastructure (eg, housing and transportation), and health care (eg, health insurance coverage).^17^ The SVM is based on zip code–level measurements of 94 SDOH variables collected directly from publicly available government survey data from the Agency for Healthcare Research and Quality (AHRQ) SDOH Database and state and city public health departments. To construct county-level SVM measures, Zip Code Tabulation Area (ZCTA) SVM estimates were aggregated to the county level using the US Census Bureau relationship files ZCTA-county and ZCTA-Metropolitan Statistical Area crosswalks based on the 2010 census.^17^ Aggregation was done by taking a population-weighted average of SVM scores across all ZCTAs assigned to a particular county. ZCTA population estimates were also obtained from US Census data. SVI and SVM data were from 2018, which were the most recent available data at the time of this analysis. All indicators for both measures were measured at the community level.

We used both social vulnerability measures because (1) they were constructed using very different statistical methodologies,^17^ (2) the SVI includes race in its construction (categories including Hispanic or Latino [of any race]; Black and African American, not Hispanic or Latino; American Indian and Alaska Native, not Hispanic or Latino; Asian, not Hispanic or Latino; Native Hawaiian and other Pacific Islander, not Hispanic or Latino; 2 or more races, not Hispanic or Latino; other races, not Hispanic or Latino), whereas by design the SVM does not, and (3) the SVI is a relative measure (national percentile) such that there will always be high and low vulnerability areas, whereas the SVM percentile is the inverse normal transform of the SVM score, and is therefore sensitive to the range of the underlying score distribution. In terms of accounting for race, we hypothesized that including race in the construction of the SVI might produce biased estimates of the association between social vulnerability and suicide rates. Regarding the difference between national percentile and a normal random variable, it may be challenging to identify specific SVI targets for interventions that would have meaningful impact on suicide rate; whereas it would be easier to develop SVM targets.

The SVI data were obtained from the CDC Agency for Toxic Substances and Disease Registry website.^16^ SVM data were obtained from the website of a test distributor (Adaptive Testing Technologies),^18^ where they are hosted and made freely available along with an app that permits visualization of their geographic distribution across zip codes and counties alongside a variety of health indicators.

County-level suicide data were obtained from the CDC WONDER (Wide-ranging Online Data for Epidemiologic Research) compressed mortality data set.^19^ The CDC WONDER data set contains county-level national mortality and population data for each year reaped from US resident death certificates and US Census Bureau estimates, respectively. The specific suicide mortality data set we used is a subset of CDC WONDER’s mortality data, in which the cause of death was determined as suicide on death certificates. The county-level CDC Wonder suicide data are suppressed by the CDC for counts of 9 or less to protect anonymity. To minimize the effect of censoring, we used 5 years of suicide data from 2016 to 2020.

### Statistical Analysis

To accommodate censoring, we analyzed the relationship between social vulnerability (SVI and SVM) and suicide using a bayesian-censored Poisson regression model, using a count of 9 as the county-level censoring threshold. We used the brms (bayesian regression models using Stan) package in R to fit the bayesian regression models, using half *t* distribution with 3 degrees of freedom as the prior.^20^ For reproducibility, we set the seed in the function to zero. To provide a nonparametric assessment of the association between SDOH and suicide (ie, to relax the assumption of a simple linear association), the SVI and SVM were categorized into 10 percentile groups (0% to 10%, 10% to 20%, etc, through 90% to 100%) and were treated as categorical variables in the analysis, with contrasts to the least socially vulnerable category (0% to 10%). As a sensitivity analysis we also modeled the SVM and SVI as having simple linear associations with suicide. The SVI provides national ordering of counties as percentiles, and therefore the 10 SVI categories have identical sample sizes. The same is not true for the SVM, for which sample sizes conform to the underlying normal distribution (Table 1).

**Table 1.** Number of Counties by Decile of SVM and SVI

Decile	Counties, No. (%)	
	SVM (N = 3141)	SVI (N = 3141)^a^
1	29 (0.9)	314
2	84 (2.6)	314
3	196 (6.2)	314
4	308 (9.8)	314
5	472 (15.0)	314
6	543 (17.3)	314
7	536 (17.1)	314
8	446 (14.2)	314
9	362 (11.5)	314
10	165 (5.3)	315

Abbreviations: SVI, Social Vulnerability Index; SVM, Social Vulnerability Metric.

^a^ Percentages not included because SVI is a relative measure arranged by decile.

Significance testing was based on incidence rate ratios (IRRs) and their 95% credible intervals (CrI). CrIs not containing the value 1.0 were considered statistically significant. To adjust for differential county population size, a population offset was added to the model. To ensure that the association between SDOH and suicide was not merely a proxy for race, age, or urban-rural county-level characteristics, the models were adjusted for county-level percentage minority status, percentage of individuals aged over 65 years in 2018, and metropolitan vs nonmetropolitan (rural) county classification,^21^ which also helped to reduce spatial correlation. We note that race was included in the SVI but not in the SVM. Percentage of individuals from racial or ethnic minority groups and those over age 65 years vary widely by county, and both are related to suicide, so we felt it important to adjust for them. Furthermore, as shown in our original SVM paper,^17^ minority status was an important moderator of social vulnerability, albeit Black individuals have lower suicide rates than White. As sensitivity analyses, we analyzed the data with and without minority status adjustment, hypothesizing that adjustment would lead to different associations with suicide for the SVI, but not the SVM. Estimated marginal suicides were then expressed in terms of annual rates per 100 000 persons. We also analyzed the association between the 4 SVI domains (socioeconomic status [SES], household characteristics, racial and ethnic minority status, and housing type and transportation) and suicide individually. Analyses were restricted to adults ages 18 years and older and used adult suicides and county-level populations only. All data were analyzed using STAN version 2.31 in R (R Project for Statistical Computing).

## Results

**Figure 1.**
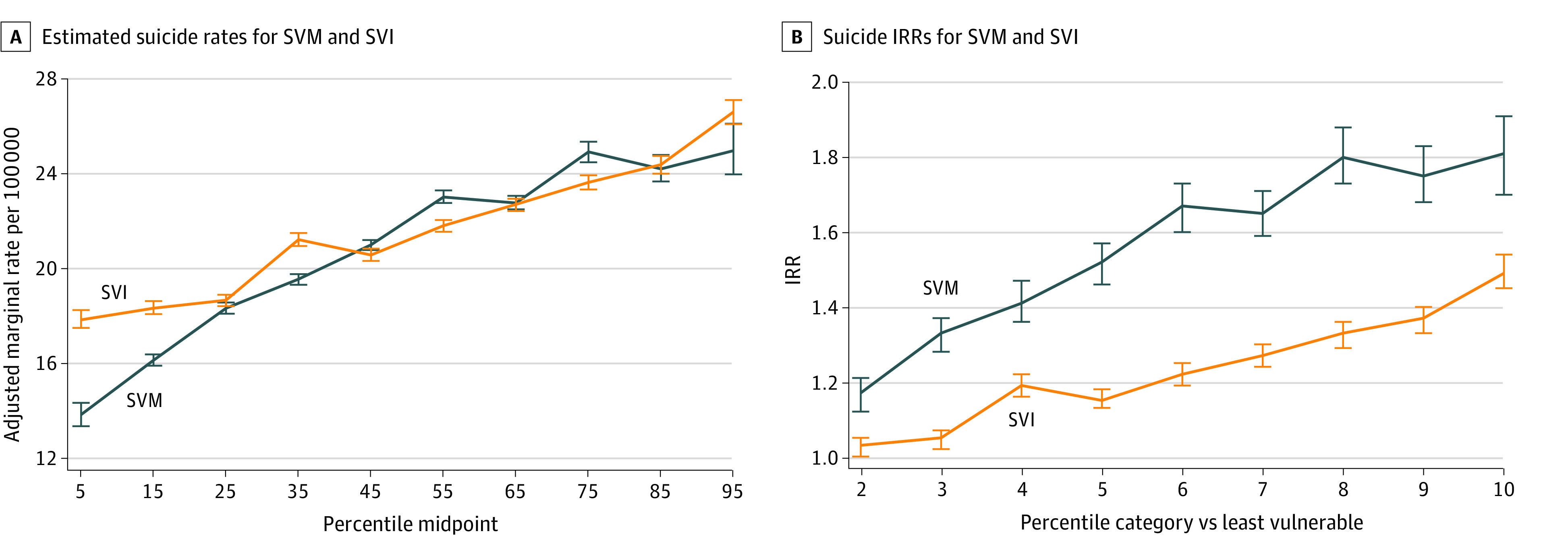
Adjusted Marginal Annual Suicide Rates per 100 000 Persons and Incidence Rate Ratios (IRR) for SVM and SVI SVI indicates Social Vulnerability Index; SVM, Social Vulnerability Metric. Whiskers represent 95% credible intervals.

There were 2401 uncensored counties and 740 censored counties. For the uncensored counties, the annual adult unadjusted suicide rate per 100 000 persons was a mean (SD) 18.3 (13.4) and a median (IQR) 19.6 (10.9-25.7) for the period of 2016 through 2020. Table 1 presents the distribution of counties across the 10 SVM and SVI intervals. Adjusted estimated marginal suicide rates per 100 000 persons are displayed in Figure 1A and Table 2 across the SVI and SVM percentile categories. Both functions are essentially linear (although not constrained to be so by the statistical model). For the SVM, suicide rates per 100 000 persons ranged from 13.8 for the least socially vulnerable to 25.1 for the most socially vulnerable. For the SVI, rates per 100 000 ranged from 17.3 for the least socially vulnerable to 27.0 for the most socially vulnerable. All IRRs for both the SVI and SVM were statistically significant (Figure 1B). Across the range of the SVM, there was an 82% increase in suicide rates (IRR, 1.82; 95% CrI, 1.72-1.92), and a 56% increase for the SVI (IRR, 1.56; 95% CrI, 1.51-1.60). When constrained to be linear (ie, SVM and SVI measures treated as continuous variables), across the range of the SVM, there was an 95% increase in suicide rates (IRR, 1.95; 95% CrI, 1.90-2.00), and a 57% increase for the SVI (IRR, 1.57; 95% CrI, 1.53-1.60). For estimated IRRs and 95% CrIs for each of the 4 SVI domain percentiles, significant positive associations were found for SES (IRR, 1.43; 95% CrI, 1.39-1.47) and household characteristics (IRR, 1.56; 95% CrI, 1.52-1.60), significant inverse association with racial and ethnic minority status (IRR, 0.82; 95% CrI, 0.79-0.85) and housing type and transportation (IRR, 0.95; 95% CrI, 0.92-0.98).

**Table 2.** Estimated Marginal Suicide Rates per 100 000 for SVM and SVI Deciles

Decile	SVM, marginal mean (95% CI)	SVI, marginal mean (95% CI)
1	13.82 (13.33-14.32)	17.83 (17.49-18.24)
2	16.12 (15.89-16.37)	18.32 (18.07-18.62)
3	18.32 (18.09-18.56)	18.66 (18.41-18.89)
4	19.55 (19.31-19.76)	21.22 (20.95-21.50)
5	21.00 (20.78-21.20)	20.57 (20.32-20.84)
6	23.02 (22.77-23.30)	21.81 (21.55-22.05)
7	22.77 (22.50-23.07)	22.71 (22.43-22.95)
8	24.93 (24.49-25.36)	23.64 (23.34-23.94)
9	24.21 (23.68-24.80)	24.39 (24.01-24.76)
10	24.98 (23.98-26.12)	26.62 (26.10-27.12)

Abbreviations: SVI, Social Vulnerability Index; SVM, Social Vulnerability Metric.

**Figure 2.**
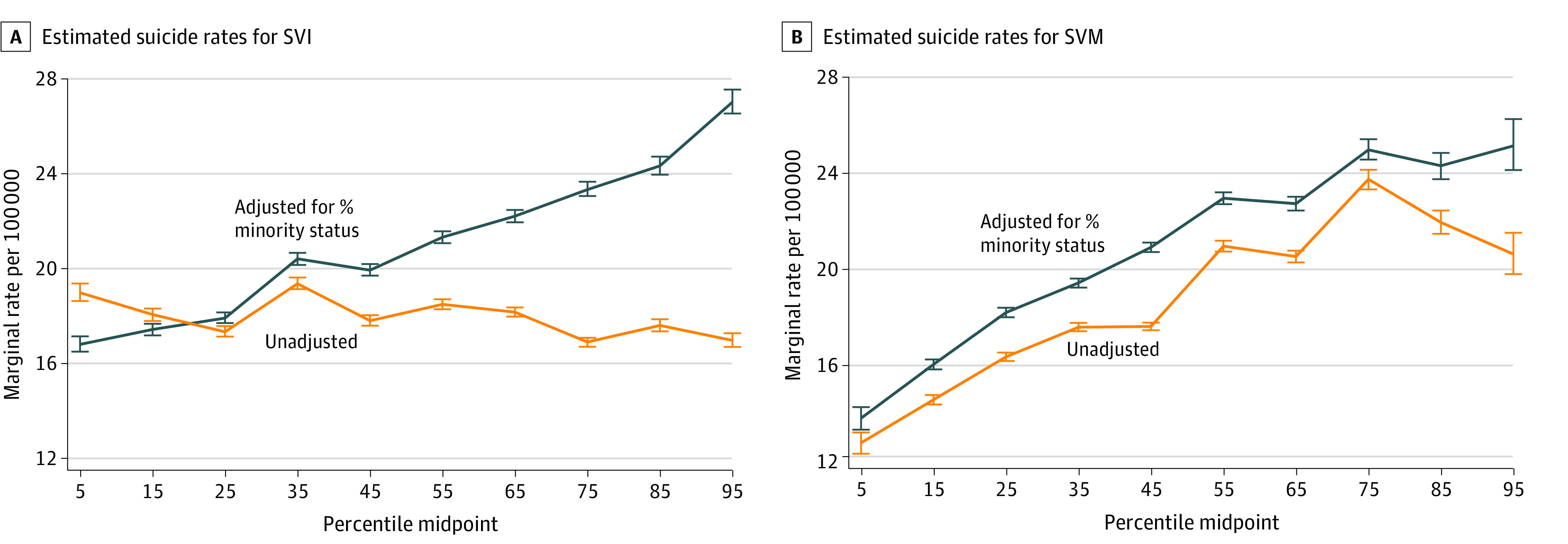
Marginal Annual Suicide Rates per 100 000 Persons for SVM and SVI Adjusted for Racial and Ethnic Minority Identity vs Unadjusted SVI indicates Social Vulnerability Index; SVM, Social Vulnerability Metric. Whiskers represent 95% credible intervals.

Across the range of the SVI, for the unadjusted model there was in fact an inverse association with suicide rate (IRR, 0.89; 95% CrI, 0.87-0.92); however, adjusting for minority status alone yields a significant positive association between the SVI percentile and suicide rate (IRR, 1.61; 95% CrI, 1.56-1.65) (Figure 2A). The same is not true for the SVM, which does not include race or ethnic minority status in its construction. For the SVM, both adjusted (for minority status only) and unadjusted percentiles have significant positive associations with suicide rate (adjusted IRR, 1.84; 95% CrI, 1.74-1.95; unadjusted IRR, 1.63; 95% CrI, 1.54-1.72), although the IRR adjusted for racial and ethnic minority status was significantly higher than the unadjusted estimate (ie, the CrIs do not overlap) (Figure 2B).

## Discussion

When adjusted for age, minority status, and urban or rural county-level characteristics, higher scores on both the SVI and the SVM were associated with significant increases in county-level suicide rates. The SVM showed a higher effect size for the association between social vulnerability and suicides, with an 82% increase (13.8 persons per 100 000 to 25.1 persons per 100 000) vs the SVI with a 56% increase (17.4 persons per 100 000 to 27.0 persons per 100 000) across their ranges. The higher point estimate seen in the SVM is consistent with earlier results for all-cause age-adjusted mortality, in which higher SVI and SVM percentiles (more vulnerable) were associated with increased mortality rates, but the SVM explained 46% of variation and the SVI explained only 12% of variation.^17^ The analysis of these data raises questions regarding the use of racial and ethnic minority status in the construction of the SVI. In unadjusted analyses, we found that increasing social vulnerability as measured by the SVI was associated with decreased suicide risk. This is because racial and ethnic minority status is associated with decreased suicide risk (Table 3), and as shown in Saulsberry et al,^17^ minority status is associated with increased social vulnerability. Statistical adjustment for minority status adjusts for the inverse association between minority status and suicide rate and yielded a significant positive association between the SVI and suicide rate. The same was not true for the SVM, which does not include racial and ethnic minority status in its construction. The SVM had a significant positive association with suicide rate, whether it was adjusted for minority status or not, although adjustment for minority status produced a greater point estimate. The association between the SVM and suicide rate was also significantly stronger than the association between the SVI and suicide rate in the adjusted analysis. The domain-specific SVI analyses suggest that the positive association between social vulnerability and suicide rate is driven largely by SES and household characteristics.

**Table 3.** Estimated Incidence Rate Ratios (IRR) for SVI Domains

Decile	SES, IRR (95% CrI)	Household, IRR (95% CrI)	Racial or ethnic minority, IRR (95% CrI)	Housing, IRR (95% CrI)
1	1 [Reference]	1 [Reference]	1 [Reference]	1 [Reference]
2	1.13 (1.11-1.15)	1.12 (1.10-1.14)	0.99 (0.96-1.03)	1.03 (1.00-1.06)
3	1.15 (1.13-1.18)	1.21 (1.19-1.23)	0.99 (0.95-1.02)	1.04 (1.01-1.07)
4	1.21 (1.19-1.23)	1.40 (1.38-1.42)	1.00 (0.96-1.03)	1.05 (1.02-1.08)
5	1.32 (1.29-1.34)	1.33 (1.30-1.35)	1.03 (0.99-1.06)	1.08 (1.05-1.11)
6	1.28 (1.26-1.30)	1.39 (1.37-1.42)	1.07 (1.03-1.10)	1.10 (1.07-1.13)
7	1.28 (1.25-1.30)	1.39 (1.37-1.42)	1.09 (1.05-1.12)	1.09 (1.06-1.11)
8	1.38 (1.35-1.41)	1.38 (1.35-1.40)	1.02 (0.99-1.06)	1.13 (1.10-1.16)
9	1.40 (1.37-1.44)	1.44 (1.41-1.47)	1.04 (1.00-1.08)	1.03 (1.01-1.06)
10	1.37 (1.34-1.41)	1.51 (1.47-1.56)	0.93 (0.89-0.96)	0.95 (0.92-0.98)

Abbreviations: CrI, credible interval; SES, socioeconomic status; SVI, Social Vulnerability Index.

Our results provide a measurable approach to reducing suicide rates through interventions that target social vulnerability.^15^ Improving access to mental health services, through changes in and availability of insurance coverage for these services, can lead to reductions in suicide rates. Decreasing social isolation by building social support networks, promoting community involvement, and reducing stigma around mental health issues can also reduce suicide rates. Furthermore, identifying those at risk for social vulnerability and suicide via approaches such as the one provided here will be critical in implementing early-prevention strategies for suicide such as therapeutic approaches and social-emotional learning and resilience programs. Increasing access to quality health care in general, particularly in rural areas by expanding Medicaid coverage for low-income individuals and families and increasing the number of health care workers in underserved areas is also likely to decrease suicide rates.

### Strengths and Limitations

The strength of this study was the use of all US suicide data over a 5-year period and 2 different validated approaches to the measurement of social vulnerability, the SVI and the SVM. There were also several limitations of this study. First, the CDC’s suppression of county-level suicides less than 10 (for privacy reasons) added uncertainty to our estimates, particularly for smaller counties. We have addressed this statistically in our modeling using a censored Poisson regression. Second, the unit of analysis, the county, mixes smaller areas (eg, zip codes or census block groups) with heterogenous social vulnerability and geographical distribution. As such, our estimates provided a more conservative estimate, lower than the true association between social vulnerability and suicide. Third, it is likely that the censored data were from smaller counties, with lower absolute suicide counts. While still nationally representative, because our model incorporated the censored data, this can lead to lower precision for estimates in less populated areas. Nevertheless, associations between social vulnerability and suicide were found. Fourth, we did not directly adjust for spatial dependence because this was not an available option in Stan. However, we did adjust for county-level urban-rural status, which should have reduced spatial dependence. Fifth, for the SVI, which is a national percentile, there will always be high and low vulnerability areas, so it may be difficult to determine absolute targets for change that would ultimately reduce suicide rates. However, as noted previously, this is not a problem for the SVM, which is sensitive to the underlying scale of measurement. Sixth, there were potential confounders of the association between social vulnerability and suicide rates. For example, areas with greater social vulnerability may also have greater access to firearms, leading to increased lethality of suicide attempts. Additionally, there is a documented lack of access to health care in rural areas, which can contribute to higher levels of lack of rescue compared with large metropolitan areas. Adjusting for urban-rural differences in our analyses addressed these issues to some extent. Previous research^15^ has found that while number of gun shops was positively associated with suicide rates in metropolitan areas, the effect size was not as high in rural areas, suggesting that access to firearms may explain some but not all of the association between social vulnerability and suicide.

## Conclusions

This cohort study showed an associated between social vulnerability and suicide. The SVM in particular provides a method of measuring social vulnerability at the zip code or county level that can be used to enhance suicide risk prediction algorithms and target social interventions that may ultimately reduce suicide.

## Notes

Supplement.
Data Sharing Statement
