# Supplementary material for: Social Vulnerability and Risk of Suicide in US Adults, 2016-2020
Source: JAMA Netw Open. 2023 Apr 26;6(4):e239995. doi: 10.1001/jamanetworkopen.2023.9995 (PMC10134005; doi:10.1001/jamanetworkopen.2023.9995)
Supplement: Supplement. — Data Sharing Statement [file jamanetwopen-e239995-s001.pdf]

Data Sharing Statement

Liu. Social Vulnerability and Risk of Suicide in US Adults, 2016-2020. *JAMA Netw Open*. Published April 26, 2023. doi:10.1001/jamanetworkopen.2023.9995

Data
Data available: No

Additional Information
Explanation for why data not available: Data used as part of this brief report were publicly accessible. The data sets can be found on the CDC WONDER website.
